# Supplementary material for: Burden of stigma among tuberculosis patients in a pastoralist community in Kenya: A mixed methods study
Source: PLoS One. 2020 Oct 15;15(10):e0240457. doi: 10.1371/journal.pone.0240457 (PMC7561176; doi:10.1371/journal.pone.0240457)
Supplement: S1 File — (DOCX) [file pone.0240457.s001.docx]

**SEMI STRUCTURED INTERVIEW GUIDE.**

(a) I would like to know about the experience you have gone through since you started ailing from Tuberculosis (TB).

(b) How did you interact with the rest of the family /community after realizing you had TB?

(c) How did those around you treat you when they knew you had TB? Probe on social support

system from family, friends, the community, the neighbors or health workers/facilities.

(d) Did you experience any form of discrimination/stigma before, during and after treatment? Why?

(e) Is there any other information regarding TB stigma you would want to tell us?

**FOCUS GROUP DISCUSSION GUIDE**

a) Let us talk about TB stigma.

b) What is it like to be a TB Patient, what kind of experience does a TB patient through?

c) Do TB patients face any form of discrimination?

d) How do TB patients interact with the rest of the family /community (probes: do they isolate themselves or are they isolated by others? how?

e) How does the family/community members expect TB patients to conduct themselves?

f) What social support do TB patients receive from family/community?
